# Home care nurses’ perception of the challenges they faced during the COVID-19 pandemic: a qualitative study

**DOI:** 10.1186/s12912-022-01082-y

**Published:** 2022-11-16

**Authors:** Tahereh Najafi Ghezeljeh, Sahar Keyvanloo Shahrestanaki, Zahra Amrollah Majdabadi kohne, Ehsan Fakhari

**Affiliations:** 1 Nursing and Midwifery Care Research Center, School of Nursing and Midwifery, Iran University of Medical Sciences, Tehran, Iran; 2 School of Nursing and Midwifery, Iran University of Medical Sciences, Tehran, Iran; 3 School of Nursing and Midwifery, Tehran University of Medical Sciences, Tehran, Iran

**Keywords:** Nurse, Challenges, Home care, Covid-19

## Abstract

**Background:**

Despite the significant role they play in the whole health care system, home care nurses are not paid the attention they deserve. Besides highlighting their significant role in the health care system, the COVID-19 pandemic also posed several challenges for home care nurses.

**Objective:**

The purpose of this study is to explain the nurses’ perception of the challenges of home care during the Covid-19 pandemic.

**Methods:**

The present study was a qualitative study with a conventional content analysis approach conducted from November 2020 to September 2021. Participants included 16 home care nurses who were purposefully selected based on the eligibility criteria. After obtaining ethical authorization, the data were collected through semi-structured interviews. MAXQDA Version 10 software was used for data mangement. Data analysis was performed using the Granheim and Lundman five-step method. Guba and Lincoln criteria were utilized for trustworthiness.

**Findings:**

The seven main categories obtained in this study included “the onset of a new chapter: from avoidance to relapse”, “burnout”, “vortex of moral distress”, “social stigma”, “difficulty in breaking the transmission chain”, “care inhibitors related to the patient and family” and “support deficiency: the crisis of home care nursing agencies during the crisis”.

**Conclusion:**

The results showed that nurses working in home care during the Covid-19 pandemic experienced several challenges in various fields. This study captured the nurses’ perception of the challenges of home care during the Covid-19 pandemic, a period of unprecedented change and difficulty. These challenges included lack of support, psychological problems, and dealing with new experiences. Identifying these challenges can help improve the quality of home care nursing and planning in this area.

**Supplementary Information:**

The online version contains supplementary material available at 10.1186/s12912-022-01082-y.

## Introduction

The covid-19 disease is spreading rapidly in the world [1, 2]. In May 2022 there were more than, 620 million people in the world infected with Covid-19 disease, of which 6.55 people had died, and in Iran, out of 7.55 million infected, 144 thousand had died by this date [3]. The widespread pandemic of Covid-19 and its consequences have affected all aspects of health services globally, including home care [2].

Home care nursing is not a new field in the healthcare system in Iran; however, recent years have seen a significant increase in the number of home care agencies. There are currently 850 home care agencies operating in the health sector and a further 1400 are awaiting the official permit [4]. At the First stage, at the discretion of the treatment team and with the consent of the families, the patient is transferred home for the care to continue [4]. After the Covid-19 pandemic in Iran, home care was divided into two general categories: caring for Covid-19 patients and caring for patients with other diseases [5]. Most people in the second group are vulnerable patients cared for at home due to some chronic diseases or complications caused by aging [6]. Home care nurses in Iran have a bachelor’s degree or higher and take courses to take care of patients at home. These nurses work under the supervision of home care agencies licensed by the Ministry of Health [7].

The Covid-19 pandemic poses many potential challenges to home care nursing due to the risk of virus transmission [8]. However, according to the literature, home care nurses have received less attention than hospital nurses during the pandemic, and a limited number of studies have been conducted on the problems and challenges faced by these people [9]. Since some studies have mentioned the high workload and vulnerability of the nursing staff in home care [10, 11], it seems that the new conditions have increased the vulnerability of these people. On the other hand, according to reports, some nurses working in hospitals have experienced safety concerns, anxiety, stress, fatigue [12, 13], and fear and anxiety caused by family members being infected with this disease [14]. Studies also show that nurses at the forefront of the fight against Covid-19 disease have suffered a great deal physically and emotionally [15, 16]. Home care nurses, like hospital nurses, seem to face many challenges. Because the home environment is designed to be lived in rather than being a place for care, these individuals face different and unique challenges [17].

A study conducted in New York indicated a variety of challenges faced by home care nurses during the COVID-19 pandemic; the challenges mentioned in this study include being ignored by other members of the community, a high chance of contracting COVID-19, a lack of training courses during the pandemic, and a shortage of protective equipment and facilities [18]. However, there are few studies conducted to inspect the challenges faced by home care nurses in Iran specifically during the COVID-19 pandemic; therefore, there is demand for further studies in this sector of Iran’s healthcare system [10]. On the other hand, home care nursing challenges during the Covid-19 pandemic in Iranian society was influenced by cultural and social factors [19]. The home care nurse usually has a close relationship with family members and the patient, so it seems that he/she has a close relationship with the social structure and family customs and has his/her own problems [20, 21]. In fact, nurses’ perceptions of the crisis are shaped by the complexities of laws, patients, families, and home care agencies [22].

A careful inspection of the faced challenges can present a clear picture of the nurses’ conditions during the COVID-19 pandemic, which will in turn result in effective measures to improve home care conditions. Therefore, the present study explains the nurses’ perception of the challenges of home care during the Covid-19 pandemic.

## Materials and methods

### Study design

The present study was performed using a qualitative method and a conventional content analysis approach based on the steps proposed by Granheim and Lundman. The conventional content analysis approach is a flexible method for analyzing textual data. It is used when there is not enough knowledge about a phenomenon and, with an inductive approach, emphasizes the creation and development of categories and the interpretation of written or spoken content. In this approach, categories and their names emerge from the data [23]. This article is based on the COREQ (consolidated criteria for qualitative research) checklist (Supplementary File 2) [24]. This study was performed in three home care nursing agencies affiliated with the Iran University of Medical Sciences, Tehran, Iran.

### Participants

Participants included 16 home care nurses who were selected through purposive sampling, and the sampling was continued until data saturation (categories saturation table is attached to the article). Inclusion criteria included more than 1 year of work experience in home care, a bachelor’s degree or higher in nursing, the experience of home care during the Covid-19 pandemic, and a willingness to participate in the study. Exclusion criteria included reluctance to participate in the study and restrictions of cooperation. None of the nurses were excluded from the study. The first person in this study was an experienced one who, after being briefed on the objectives of the study and how to participate, entered the study willingly.

### Data collection

This study began in November 2020 after receiving the code of ethics and ended in September 2021. A semi-structured interview method was used to collect data. The interview guide was prepared and approved based on three pilot interviews. Interview times ranged from 30 to 60 minutes. The Place for the face-to-face interview was chosen according to the participant’s preference: in one of the rooms of the home care agencies. Examples of questions used in the interviews were: “How would you describe home care for people with Covid-19?”, “In your experience, what are the challenges and problems in caring for a patient at home during the Covid-19 pandemic?” All the interviews ended with this question: “Is there a question that came to your mind but I did not ask?” Or “Is there anything else you want to add?” Due to the outbreak of Covid-19,causing less contact with the participants, some of the interviews were conducted through WhatsApp or Skype. The interviewer was experienced in conducting interviews for qualitative research. With the participants’ permission, all interviews were recorded by a recorder and then typed word by word by the interviewer. MAXQDA 10 series software was used to manage the data while maintaining the confidentiality of the information. Further interviews were performed with the participants 1–2–4-7-10 to increase the trustworthiness and eliminate some ambiguities or questions. No new data were collected in these interviews and all the concepts seemed to be well defined and explained (Supplementary File 1). Then, to ensure saturation, two additional interviews were conducted beyond saturation, and the data of these people were placed in categories.

### Data analysis

Data analysis was performed simultaneously with data collection through Graneheim and Lundman (2004) steps (Table 1) [23]. The interviews were immediately recorded and transcribed. The transcripts of the interviews were read several times to immerse the researcher in the data. The words, sentences, and paragraphs relevant to the purpose of the study in each interview were considered meaning units. These meaning units were coded using participant words or appropriate tags extracted from the data. The codes were constantly reviewed and compared. Similar items were placed in common categories and then the main categories were created.

**Table 1:** Steps of data analysis by Graneheim and Lundman (2004) method

	stages	Description of actions in each step
1	Collecting data	Conducting face-to-face and in-person interviews, conducting complementary interviews, an audio recording of interviews
2	Word-by-word transcript of each interview	Frequent listening to interviews and transcribing it word by word
3	Reading the text of the interview to understand its main content and to extract the meaning units	Re-reading the content to gain a general and in-depth perception of the participants’ statements and extract the meaning units
4	Determining and coding the meaning units	Examining participants’ explanations and determining meaningful sentences and producing codes
5	Classification of primary codes	The obtained concepts were classified into specific categories (based on the similarity of the concepts).
6	Identify the content hidden in the heart of the data	Extracting important explanations and giving meaning to them with unique concepts (expressing the meaning of essential parts) and finally clearly and unambiguously expressing the challenges of nurses in the study.

### Trustworthiness

The trustworthiness of the data in this study was evaluated based on the steps proposed by Lincoln and Guba [25]. The member checking method was used to ensure credibility, during which, home care nurses were asked to confirm the conformity between the categories produced and their experiences. Two experts also used peer checking in the field of qualitative studies for the trustworthiness of data coding and classification. Scrutiny of the data and the related documents was conducted by two external observers and the preservation of documents related to the different stages of the research added to the dependability of the study. To ensure transferability, the researcher gave detailed explanations, such as a detailed description of the participants, the sampling method, and the time and place of data collection, so that the reader could comment on the transferability of the findings. Recording all the procedures of the study and reporting the research process enhanced the confirmability.

### Findings

A total of 16 nurses were interviewed. The mean age of the participants was 31.25 ± 3.60. Demographic characteristics of the participants are reported in Table 2. The main findings of this study included 7 categories and 22 sub-categories (Table 3). Main Categories included the onset of a new chapter: from avoidance to relapse, burnout, the vortex of moral distress, social stigma, difficulty in breaking the transmission chain, care inhibitors related to patient and family, and deficiency of support: the crisis of home care nursing agencies during the crisis. It should be noted that due to the number of times problems were expressed by nurses, study participants understood all the challenges equally and gave equal importance to all the challenges.

**Table 2:** Demographic characteristics of the participants

No.	Gender	Age	Marital status	Education	Working shift	Work experience in-home care (year)	Clinical work experience in hospital (year)
P1	Male	32	Single	Masters	Mix of day and night	6	12–13
P2	Male	30	Single	Masters	Night	5	9
P3	Male	36	Single	Bachelor (Supervisor)	Mix of day and night	9	15
P4	Male	31	Single	Masters	Mix of day and night	4	8
P5	Male	40	Married	Masters	Mix of day and night	11	15
P6	Male	31	Single	Masters	24-OFF	4	10
P7	Male	28	Married	Masters	Mix of day and night	2	4
P8	Female	27	Married	Masters	Mix of day and night	2	6
P9	Female	29	Single	Masters	Mix of day and night	3	5
P10	Female	30	Married	Masters	Mix of day and night	4	9
P11	Male	28	Single	Master (Supervisor)	Mix of day and night	6	7
P12	Female	31	Married	Masters	Mix of day and night	9	10
P13	Female	30	Single	Masters	Mix of day and night	4	8
P14	Female	35	Single	Masters	Mix of day and night	14	5
P15	Male	35	Single	Masters	Mix of day and night	15	9
P16	Female	27	Married	Masters	Mix of day and night	2	6

**Table 3:** Categories, sub-categories, and some quotes of the participants

Category	Sub-Category	Participants’ statements
The onset of a new chapter: from avoidance to relapse	Facing emerging developments	“Before the Covid-19 outbreak, we lived with sick family members and commuted with them normally, but suddenly everything seemed to change, and all our routines changed. It was as if our home nursing was divided into pre-corona and post-corona eras. Everything changed suddenly. And we faced a new and unknown disease “(P8).
	leaving the care place	“When Covid-19 came early, I had no desire to continue working in home care at all. When they called me and asked me to help the patient, I refused” (P1).
	Re-orientation and gradual return	“Step by step, it became normal for us to go to the patient’s house for care. We were not scared anymore. Step by step, we realized that the Covid-19 is less transmitted through the surface and most of its transmission is respiratory. For example, we no longer needed to disinfect our whole head and body. We went to the patients’ homes more easily for care “(participant 3). “Well, in the beginning, our income was very low, but well, because they felt the need to raise salaries, the income gradually increased. We also needed money, and step by step, we accepted and returned” (P2).
Burnout	Mental pressure due to vulnerability	“We were very scared and anxious at the very beginning of Covid-19. We were all afraid of getting Covid-19, and since I did not know anything about it, we thought that God knew what would happen to us later” (P7).
	Physical injury	“I thought I had gotten Covid-19 because of the long shifts and fatigue. At first, it was accompanied by hoarseness. Then, at night I went to rest. I had a high fever. From the third and fourth day onwards, I lost my sense of smell and taste. It lasted for a month and a half. I had severe shortness of breath and was hospitalized in the ICU for a few days” (P8).
	Stress caused by injury	“Well, we have a family. They usually wonder if it has happened to me, it was because of what I accepted to do, but what about my family? I don’t want anything to happen to my family” (P13).
The vortex of moral distress	Spiritual suffering	I always had a pang of conscience about being a vector because one of my family members also got covid-19. I told myself that I must have transmitted the disease to him “(P15). “I was ashamed of myself for being so ignorant. I felt guilty that the drugs we gave patients didn’t work” (P10).
	Resulting helplessness	“I’ve felt useless since Covid-19 came. I feel bad about telling my family that I cannot do anything else. Take your patients to the hospital not to get worse. It seems that this virus and its mutations are not over, and it does not want to give up on us. You know, the Covid-19 has become like a vortex, where you have to sink more, and you will not get anywhere “(P14).
Social stigma	Perceived stigma	“Everyone, even our own families, was afraid that we would be vectors. In the patient’s house, if something got into your throat and you coughed, that was enough; they thought you had Covid-19. You couldn’t prove that it was just something that got into your throat and nothing else.(P2)
	Perceived discrimination (feeling ignored)	“We, the home nurses, were oppressed during the Covid-19. Everyone on TV was talking about the hospital nurses. No one named us at all. At the time of vaccination, we were included in the last group of medical staff who were vaccinated “(P10).
	Perceived rejection	“Many of us did not say at all that we were working in the ward, especially to the families of the patient at home. If we had said it, they wouldn’t have let us go to their house at all “(P11). “For example, you used to go to a vegetable shop and say ‘I am a nurse’ then they would treat you very respectfully, but from the moment Covid-19 came, they ran away from us.’ “Once a taxi driver found out that I was a nurse and was working in the Covid-19 ward, he dropped me off for fear of getting the disease” (P12).
Difficulty in breaking the transmission chain	Physical separation difficulties	“As soon as we entered the house, we tried to isolate the environment where we were supposed to stay. For example, I told the family to prepare a separate room to take care of the patient and leave the patient there. “I also had to stay in the patient’s room a lot if they had Covid-19 disease; or if the patient was ill and we had to stay with him all the time, I would try to open the windows so that there was enough ventilation, but this was not possible in every house.” P4).
	Difficulty implementing personal protection strategies	“Especially in those early days, it was very difficult for us to bring food, dishes, spoons, and forks from home. It was very difficult to stay in the protective clothing in that house with a twenty-four-hour shift, we were constantly sweating, and we were in trouble” (P6).
	Gradual decline in following the protocols over time	“In the beginning, we were very careful; we were constantly washing our hands and our hands’ skin was always cracked, and the patient’s family disinfected me as soon as I got home; they even disinfected my backpack. But now it’s not like then. Both we and our family just wear masks, because we have been vaccinated; well, we seem to be less careful” (P5). “Step by step, we learned that the disease is not transmitted through surfaces and it is more respiratory, and it is enough if we just put on a mask, as if we are not scared anymore and it is normal for us” (P1).
Care inhibitors related to the patients and their families	Family-related care inhibitors	“Every time we told the family that wearing these procedure masks was vital for your patient, they would say, ‘No, my patient is being bothered’ and he would come and take off the patient’s mask” (P12). “The family tried everything they read on the Internet on the patient (with Covid-19 disease). Or they gave the patient some unknown kind of herbal drink even if we said it could be harmful to the patient and that they had to consult a doctor.(P9). “Once I saw a patient and accidentally found out that he had Covid-19 and the family hadn’t told the agencies and us about it” (P1).
	Patient-related care inhibitors	“Taking care of Covid-19 patients at home was very difficult because I saw several new symptoms and complications from the patient that we had not encountered before. For example, most of these patients had cognitive problems and did not cooperate with the nurse” (P6). “Most Covid-19 disease patients were terrified because their disease was unknown, and they were all scared of dying” (P3).
Lack of support: crisis of agencies in crisis	Crisis mismanagement	“In my opinion, the nursing home care agencies in my home town during the pandemic were very poorly managed. They passed a new and hasty law every day and told us to exercise it. Suddenly, they called us and told us to go on an extra shift, otherwise they would cut off their cooperation with us” (P13).
	Lack of supervisor competence	“The supervisor plays a vital role in helping the nurses. If I have a problem somewhere, I can get help from her. We did not know much in Covid-19. For example, I did not know how to work with the BiPap, but our supervisor was not very good either “(P8). “The supervisor must be able to communicate well with the patient’s family. Many of them could not cope” (P1).
	Lack of information support: Lack of comprehensive training	“We did not have any training program in Covid-19. The previous training we had was also canceled during the pandemic and I did not receive any training at all. This increased our fear and anxiety at work and I was afraid of doing the procedures the wrong way. Or I was afraid of getting COVID-19 because I didn’t know how to protect myself”(P9).
	Lack of financial and legal support: No compensation for inefficient services	“Our payment was very, very low. The same small amount of salary was paid with a total delay. I once got Covid-19and took a sick leave. They told me that I could not be absent for more than three days and that I had to return to my job “(P14).
	Lack of logistical support: drug and equipment shortages	“Insurance did not cover home care. All equipment had become very expensive and scarce. For example, nursing care agencies had rationed masks and gloves for us” (P11).
	Lack of support for efficient human resources: Human resource mismanagement	“Early on, doctors were either busy in hospitals or were too scared to visit homes. Physiotherapists also did not come to the patient. The patient’s family begged them but they did not come “(P4). “The number of nurses in the house was so low that we had to take the whole burden. On the other hand, because there was inadequate human resources, the nursing care agencies started hiring nurses who had no experience in home care and with no training “(P16).

*P* Participant

### The onset of a new chapter: from avoidance to relapse

From the interviewees’ point of view, one of the challenges of home care in the Covid-19 pandemic was the onset of a new chapter: from avoidance to relapse, which included three sub- categories: dealing with emerging developments, vacating the field of care, and a gradual re-orientation to care. Participants noted that before the outbreak of Covid-19, they experienced normal conditions in home care and normally lived with the patient’s family and took care of the patient. The emerging changes occurred suddenly and they encountered an emerging and unknown disease. The nurses pointed out that at the beginning of the outbreak, they did not tend to take care of the patients at home personally, and some of them even refused to take care of patients at home and withdrew from care. The nurses also noted that they gradually returned to care over time.

“Everything suddenly changed, the sudden arrival of Covid-19 changed all our routines and plans. I was not the only one who refused to go home to take care of the patient. Many of my colleagues left home care and resigned. But as time passed, we started taking care of the patients again ”(P10).

### Burnout

From the nurses ‘perspective, the second challenge of home care during the Covid-19 pandemic was burnout, which consisted of the mental pressure due to vulnerability, physical injury, and the stress caused by injury. According to the participants, burnout was one of the most critical and influential consequences of the pandemic, which affected the care and the lives of nurses working in the home care sector. Mental pressure due to vulnerability in nurses during the Covid-19 period took on wide dimensions such as anxiety, fear, worry, depression, and a sense of insecurity. Many of them experienced a constant feeling of uncertainty about the environmental pollution or the possibility of the patient and the patient’s family getting Covid-19. Participants stated that due to the lack of nurses in home care and the family’s request to reduce commuting to the home, nurses were subjected to long shifts and experienced high degrees of physical fatigue, leading to Covid-19 disease and severe symptoms and complications. Many nurses expressed great fear and concern for others and their families (major concerns).

“It is fear and anxiety on one hand and its physical fatigue on the other. We believe it is part of our job but we also have a family. We usually say that if it happened to me, it is because of what I accepted to do, but what about my family? I don’t want anything to happen to my family”(P14).

### Vortex of moral distress

From the participants’ point of view, another critical challenge in home care was the vortex of moral distress. The moral distress further led to helplessness and feelings of futility. According to the participants, spiritual torture and the resulting helplessness were the essential components of the vortex of moral distress. The nurses stated that they felt ashamed and had the pang of conscience, guilt, helplessness, disability, and futility in the face of the disease.

The nurses even likened the Corona virus and its mutations to a vortex they had been sucked into and which they could not escape.

“Hearing people die and suffer made me feel guilty because I saw that people were still getting sick despite the vaccine. I was embarrassed that no medicine was efficient and that the patient was getting sick and we were sending him to the hospital. I was feeling helpless and completely paralyzed because of Covid-19 and its endless mutations. I felt that my nursing skills no longer worked ”(P16).

### Social stigma

From the participants’ perspective, social stigma was one of the challenges of home care during the Covid-19 pandemic. One of the challenges that nurses experienced during this period was the suspicion of those around them about being a vector. The nurses also stated that they were being ignored compared to the hospital nurses so even the vaccination of these people was delayed compared to the hospital nurses.

They were even isolated due to the suspicion that they had contracted the disease and that they were acting as vectors. This isolation caused them to hide their nursing profession in the community.

“If anyone in the family knew you were a home care nurse of a Covid-19 patient, they ran away. They always thought you were a Covid-19 vector. In the society, if someone knew that you were the home care nurse of covid-19 patients, they would not take you in a taxi. I was lonely and so I walked away. We were one of the last groups to be vaccinated; but we also work with the Covid-19 patients, so what was the difference between the hospital nurses and us? ”(P3).

### Difficulty in breaking the transmission chain

Nurses took steps to prevent members involved in care from transmitting the Covid-19 virus and tried to break the transmission chain. The difficulty in breaking the transmission chain was one of the challenges of home care during the Covid-19 period for participants. One of the challenges the nurses faced in breaking the transmission chain was physical separation. To do this, nurses had to develop strategies in the confines of the home to isolate their presence, the patient, and the equipment. On the other hand, the implementation of the personal protection strategies had many difficulties (lack of equipment, difficulty of observance, and exhaustion) in the long shifts. Despite the many efforts of nurses in implementing these strategies, over time, nurses saw a gradual decline in following health protocols and the problems associated with them.

“We had to isolate ourselves from the rest of the members in the house. You know, the situation in the house changes completely due to its limitations. You know wearing those clothes in the house where we used to have comfortable clothes was a separate issue, but these were for earlier times. Now, it’s not like that anymore. Both we and the family only wear masks and can no longer dress like before” (P11).

### Care inhibitors related to patient and family

Care inhibitors related to patient and family included family-related care barriers and patient-related care barriers. The nurses stated that some families were unable to provide adequate equipment for their patients in some cases due to shortages or high prices.

Furthermore, some other families who believed in traditional medicine insisted on carrying out some traditional customs such as visiting the patient in groups or giving him/her medicinal plants. Also, some families trusted whatever medical advice they read on the internet and insisted on carrying it out on the patient.

Nurses facing the challenge of patient-related care barriers pointed to non-adherence to treatment due to cognitive deficits, high fear of Covid-19 infection and death, as well as the occurrence of various problems and complications.

“The families behaved very emotionally. They did not think they were getting the Covid-9 themselves now; they kept coming to see the patient and did not keep their distance. The patient was restless, and we weren’t allowed to take care of him/her. When they met the family, they looked worse, and the family wanted to test everything they heard from here and there on the patient ; Things such as herbal medicine and other things ”(P5).

### Lack of support: crisis of home care nursing agencies during the crisis

‘Lack of support; the crisis of home care nursing agencies during the crisis was another challenge related to home care in the Covid-19 pandemic. One of the challenges for nurses in home care was the crisis mismanagement of the agencies. Instead of crisis management, the agencies fueled the problems and the challenges of this period with mismanagement and lack of planning and anticipation. On the other hand, according to the nurses, the supervisors, who had an important role in controlling and managing the crisis as an observer, were not sufficiently qualified in this field. Nurses announced they faced a lack of training despite the need for up-to-date training. In addition to all these challenges, nurses pointed to the lack of financial and legal support (untimely and inadequate payment, not exercising the labor law, not being given a sick leave, and not exercising the insurance law). Lack of logistical support (drug and equipment shortages) was another challenge for home care nurses, who, in some cases, rationed personal protective equipment. In addition to the lack of equipment, nurses in the Covid-19 crisis faced the challenge of lacking efficient human resources (doctors, nurses, and medical teams).

“Our agencies were so badly managed that it looked like there was no crisis. The equipment was rationed for us. We did not have gloves or a syringe. They were constantly increasing our shift hours. “Whatever we said to the supervisor, she could not manage, she was only the victim of his seniors” (P12).

## Discussion

This study was the first of its kind studying the challenges faced by home care nurses during the COVID-19 pandemic in Iran. The findings of this study indicate that these nurses experienced a new season in their job during this period. Being afraid of contracting the disease at the beginning of the period, home care nurses refrained from fulfilling their duties at the beginning of this period. However, they gradually returned to their jobs. The two major reasons that made home care nurses return to their jobs was first, their financial needs, and second, the support people and the government started to give them midway through the pandemic. This support showed itself in titles such as “health care heroes” and “health care warriors” which were given to the health care staff. Unlike home care nurses in Iran, their counterparts in other countries [26] and in hospitals in Iran [27] didn’t discontinue their jobs at the beginning and continued looking after patients into the pandemic. This study indicates that these nurses lacked adequate training and preparation to face a crisis; therefore, they stepped away from fulfilling their duties due to the fear of contracting the disease. The participants in this study stated that home environment was not infection-free compared to that of a hospital. They also believed that home care shifts were twice as long as hospital shifts and this increased chances of contracting the disease. The nurses in this study also stated that they were not able to disinfect the home environment in a proper way and therefore refused to do their jobs at the beginning of the pandemic. Proper training is a contributing factor in nurses’ crisis management ability and infection control [20] and numerous studies have indicated a lack of regular training for home care nurses [20]; therefore, creating and implementing a scheme that prepares home care nurses for new diseases, trains them on how to face new crises, and instructs them in infection control is highly recommended.

In the present study, the burnout and fatigue that the nurses experienced showed themselves in the form of physical and psychological distress. The psychological distress showed itself in the form of stress and fear of contracting the disease as well as worrying about transmitting the disease to the other members of their families. Other studies have also pointed out the fear nurses had of transmitting the disease to the family members [18, 28]. Similar to the findings of other studies, the participants in the present study also experienced different levels of fear, stress, obsession, and depression during the pandemic [29, 30]. This study also confirms the results of other studies [27] indicating that fatigue results from physical and psychological burnout. Fatigue also caused negligence in safety measures to control infection, which significantly reduced patient care [31] and even threatened the patients’ as well as nurses’ health causing them to eventually contract the disease [27]. This finding also confirmed other studies’ results [26, 27]. Based on these results, it seems that the physical and psychological distress inflicted on home care nurses during a pandemic must be regularly checked in order to prevent the consequences [32].

The participants in this study also underwent some spiritual suffering and experienced feelings of helplessness. There was a unique sense of moral confusion among the participants in the present study. Some factors making home care nurses undergo moral and spiritual distress were the increasing numbers of patients along with their worsening health conditions, high death rates, the long period of the pandemic, and inability in infection control. The participants described such circumstances as a “swallowing whirlpool which left them no way to escape”. Although studies conducted in China and some Middle Eastern countries [33, 34] indicated a similar feeling of guilt and shame among nurses, such feelings appeared to be more intense among Iranian nurses [35].

Home care nurses also experienced social stigma, discrimination, and isolation during this period. Social stigma and isolation came from the circle of people close to these nurses who suspected that they had contracted the disease and therefore acting as vectors. Home care nurses also felt discriminated against as opposed to hospital nurses, who received more media attention and were more appreciated by the public during the pandemic. There are other studies confirming this finding as well [18, 28]. The social stigma attached to home care nursing could also affect these nurses’ full-hearted commitment to their social service [36]. As people highly likely to be transmitting the disease, hospital nurses also felt the social stigma attached to their jobs [37]. Home care nursing acted as support for the health care system during the pandemic and removed the pressure on the system during the periods when the number of patients increased dramatically [5]. This fact, nevertheless, didn’t change the social negligence that these nurses experienced compared to hospital nurses whose jobs received more attention during the pandemic [18].

Despite their numerous problems, these nurses did their best to adhere to protective measures; however, this protection decreased dramatically over time. The reason for this decrease was the nurses’ and the families’ decreasing sensitivity to COVID-19 over time. Similar to other studies [15, 24], this study also showed that home care nurses had a shortage of equipment. The reason for this shortage was the less attention that the society paid to these nurses compared to other nurses [18]. The study also found out that in some occasions, these nurses used their personal budget to buy the equipment. Since infection control is less possible in the home environment compared to a hospital, protective measures must be taken more seriously at home [4]; therefore, it is recommended that the supervising organizations inspect and evaluate home care agencies more carefully and promptly provide them with sufficient protective equipment.

A unique challenge indicated in this study was the role of protection inhibitors. The Iranian culture highly values family bonds, which makes Iranian people more willing to take care of their family members at home [25]. However, families are not educated properly on home care and its requirements [26]. Another observation made by this study was the families’ undue interventions, their insistence on conducting some unscientific or traditional protective measures, which caused trouble for home care nurses. Not being properly educated by professionals, families believed the information on social media and insisted on practicing them at home and on their patients. As several studies have indicated, insisting on conducting unscientific protective measures stems from the family members’ compassion towards their other family members [27].

Resisting treatment and an extreme fear of COVID-19 and its unknown consequences were among the inhibitors in this study. As a person involved in the process of treatment, the patient’s cooperation with the nurse can enhance the quality of the protection [38]. Therefore, in order to prevent such challenges, it is highly recommended that home care agencies hold training courses for both nurses and families prior to the home care process [38].

Another challenge that the nurses faced was the agencies’ mismanagement and their inability to manage the crisis. One of the reasons for this mismanagement was that home care agencies experienced a level of shock when faced by the pandemic. The agencies’ unpreparedness for the pandemic created several challenges for home care nurses. Another challenge putting these nurses under great pressure was the shortage of health care staff during the various surges in Corona cases. The agencies also failed to provide them with sufficient equipment, which in turn increased the pressure on home care nurses. Other studies also have indicated the shortage of protective equipment available to the health care staff [4, 22]. This study, along with other studies [18], indicated that home care agencies lacked reserve equipment to face the crisis, which in turn created another crisis during the pandemic. The results also showed that in numerous cases, nurses could not use their medical leave of absence because they had to return to their jobs due to a shortage of medical staff and this increased the nurses’ vulnerability to the disease [26]. These results also indicated that the agencies’ inability to manage the situation even intensified the crisis. Since crisis management is vital in controlling critical situations [28], there must be some schemes implemented prior to the crisis in order to control it at the right time [29].

It must be noted that home care is completely a new sector in Iran, and despite its remarkable improvements, there is still room for planning and improvement [39].

One of the limitations of this study was the difficulty in finding samples due to the Covid-19 pandemic. This limitation was solved by making the necessary arrangements with several different home care services agencies. Also, due to the possibility of infection and also because of the face masks, it was not possible to see the full face of the participants. Therefore, the level of respondents’ trust in the interviewer could not be determined.

## Conclusion

The findings of this study showed that nurses have experienced several challenges during the Covid-19 pandemic since 1398 (2019), which has greatly impacted the nurses’ health and how their services at home. This study captured the nurses ‘perception of the challenges of home care during the Covid-19 pandemic, a period of unprecedented change and difficulty. Based on the findings of this study, it is recommended that subsequent applied and in-depth research be conducted in order to develop comprehensive guidelines for monitoring home care nursing in the country under conditions such as the Covid-19 pandemic. Based on the findings of this study, it can be stated that identifying the challenges of home care and seeking effective and efficient solutions can help policy-making and decision-making of officials by improving the quality of information and reducing stress and anxiety, which will result in high-quality performance in critical situations.

## Supplementary Information

**Additional file 1: Supplement Table1.** Saturation grid.

**Additional file 2: Supplement Table2.** Consolidated criteria for reporting qualitative studies (COREQ): 32-item checklist.

## Data Availability

All data and additional data files in Persian are available from the corresponding author on reasonable request.

## References

[1] Wilder-Smith A, Chiew CJ, Lee VJ (2020). Can we contain the COVID-19 outbreak with the same measures as for SARS?. Lancet Infect Dis.

[2] Cheng AC, Williamson DA (2020). An outbreak of COVID-19 caused by a new coronavirus: what we know so far. Med J Aust.

[3] World Health Organization (2022). Coronavirus disease (COVID-19) Situation Report.

[4] Masoumi N, Hosseinzadeh M, VanSon C, Ghezeljeh TN (2021). Home healthcare in Iran: a hybrid concept analysis. Iranian J Nurs Midwifery Res.

[5] Raoofi A, Takian A, Haghighi H, Rajizadeh A, Rezaei Z, Radmerikhi S (2021). COVID-19 and comparative health policy learning; the experience of 10 countries. Arch Iran Med.

[6] Vandiver T, Anderson T, Boston B, Bowers C, Hall N (2018). Community-based home health programs and chronic disease. Prof Case Manag.

[7] Reckrey JM, Tsui EK, Morrison RS, Geduldig ET, Stone RI, Ornstein KA (2019). Beyond functional support: the range of health-related tasks performed in the home by paid caregivers in New York. Health Aff.

[8] Keyvanloo SS (2020). “Letter to editor” challenges of home care during the COVID-19 outbreak. Iran J Nurs.

[9] Atashi A, Nejatian A (2020). Challenges of home health care during COVID-19 outbreak in Iran. Int J Community Based Nurs Midwifery.

[10] Landes SD, Weng SS (2020). Racial–ethnic differences in turnover intent among home health aides. J Appl Gerontol.

[11] Stone R, Wilhelm J, Bishop CE, Bryant NS, Hermer L, Squillace MR (2017). Predictors of intent to leave the job among home health workers: analysis of the national home health aide survey. The Gerontologist.

[12] Karimi Z, Fereidouni Z, Behnammoghadam M, Alimohammadi N, Mousavizadeh A, Salehi T (2020). The lived experience of nurses caring for patients with COVID-19 in Iran: a phenomenological study. Risk Manag Healthc Policy.

[13] Ness MM, Saylor J, Di Fusco LA, Evans K (2021). Healthcare providers’ challenges during the coronavirus disease (COVID-19) pandemic: a qualitative approach. Nurs Health Sci.

[14] Bohlken J, Schömig F, Lemke MR, Pumberger M, Riedel-Heller SG (2020). COVID-19 pandemic: stress experience of healthcare workers-a short current review. Psychiatr Prax.

[15] Liu YE, Zhai ZC, Han YH, Liu YL, Liu FP, Hu DY (2020). Experiences of front-line nurses combating coronavirus disease-2019 in China: A qualitative analysis. Public Health Nurs.

[16] Leng M, Wei L, Shi X, Cao G, Wei Y, Xu H (2021). Mental distress and influencing factors in nurses caring for patients with COVID-19. Nurs Crit Care.

[17] Chase J-AD, Russell D, Rice M, Abbott C, Bowles KH, Mehr DR (2020). Caregivers’ experiences regarding training and support in the post-acute home health-care setting. J Patient Exp.

[18] Sterling MR, Tseng E, Poon A, Cho J, Avgar AC, Kern LM (2020). Experiences of home health care workers in new York City during the coronavirus disease 2019 pandemic: a qualitative analysis. JAMA Intern Med.

[19] Navab E, Negarandeh R, Peyrovi H (2012). Lived experiences of Iranian family member caregivers of persons with Alzheimer’s disease: caring as ‘captured in the whirlpool of time’. J Clin Nurs.

[20] Audrey S, Procter S (2015). Employers’ views of promoting walking to work: a qualitative study. Int J Behav Nutr Phys Act.

[21] Muller AE, Hafstad EV, Himmels JPW, Smedslund G, Flottorp S, Stensland SØ (2020). The mental health impact of the covid-19 pandemic on healthcare workers, and interventions to help them: a rapid systematic review. Psychiatry Res.

[22] Lang A, Toon L, Cohen SR, Stajduhar K, Griffin M, Fleiszer AR (2015). Client, caregiver, and provider perspectives of safety in palliative home care: a mixed method design. Saf Health.

[23] Graneheim UH, Lundman B (2004). Qualitative content analysis in nursing research: concepts, procedures and measures to achieve trustworthiness. Nurse Educ Today.

[24] Tong A, Sainsbury P, Craig J (2007). Consolidated criteria for reporting qualitative research (COREQ): a 32-item checklist for interviews and focus groups. Int J Qual Health Care.

[25] Lincoln YS, Guba EG (1986). But is it rigorous? Trustworthiness and authenticity in naturalistic evaluation. New Dir Prog Eval.

[26] Smeltzer SC, Copel LC, Bradley PK, Maldonado LTD, D Byrne C, Durning JD (2022). Vulnerability, loss, and coping experiences of health care workers and first responders during the covid-19 pandemic: a qualitative study. Int J Qual Stud Health Well Being.

[27] Moradi Y, Baghaei R, Mollazadeh F (2021). Protective reactions of ICU nurses providing care for patients with COVID-19: a qualitative study. BMC Nurs.

[28] Seshadri S, Concannon C, Woods JA, McCullough KM, Dumyati GK (2021). “It’s like fighting a war with rocks”: nursing home healthcare workers’ experiences during the COVID-19 pandemic. Infect Control Hosp Epidemiol.

[29] Cabin W (2021). Pre-existing inequality: the impact of COVID-19 on Medicare home health beneficiaries. Home Health Care Manag Pract.

[30] Chan EYY, Gobat N, Kim JH, Newnham EA, Huang Z, Hung H (2020). Informal home care providers: the forgotten health-care workers during the COVID-19 pandemic. Lancet.

[31] Bandini J, Rollison J, Feistel K, Whitaker L, Bialas A, Etchegaray J (2021). Home care aide safety concerns and job challenges during the COVID-19 pandemic. New Solut.

[32] Markkanen P, Brouillette N, Quinn M, Galligan C, Sama S, Lindberg J (2021). “It changed everything”: the safe home care qualitative study of the COVID-19 pandemic’s impact on home care aides, clients, and managers. BMC Health Serv Res.

[33] Kim Y (2018). Nurses’ experiences of care for patients with Middle East respiratory syndrome-coronavirus in South Korea. Am J Infect Control.

[34] Xiang Y-T, Yang Y, Li W, Zhang L, Zhang Q, Cheung T (2020). Timely mental health care for the 2019 novel coronavirus outbreak is urgently needed. Lancet Psychiatry.

[35] Pishghadam R, Esfahani FP, A. (2020). The dominance of shame or sin-oriented culture in the Iranian society. J Iran Cult Res.

[36] Bhattacharya P, Banerjee D, Rao TS (2020). The “untold” side of COVID-19: social stigma and its consequences in India. Indian J Psychol Med.

[37] Fontanini R, Visintini E, Rossettini G, Caruzzo D, Longhini J, Palese A (2021). Italian nurses’ experiences during the COVID-19 pandemic: a qualitative analysis of internet posts. Int Nurs Rev.

[38] Sama SR, Quinn MM, Galligan CJ, Karlsson ND, Gore RJ, Kriebel D (2021). Impacts of the COVID-19 pandemic on home health and home care agency managers, clients, and aides: a cross-sectional survey, march to June, 2020. Home Health Care Manag Pract.

[39] Kianian T, Lotfi M, Zamanzadeh V, Rezayan A, Hazrati M, Pakpour V (2022). Exploring barriers to the development of home health Care in Iran: a qualitative study. Home Health Care Manag Pract.

