# Supplementary material for: Home care nurses’ perception of the challenges they faced during the COVID-19 pandemic: a qualitative study
Source: BMC Nurs. 2022 Nov 16;21:314. doi: 10.1186/s12912-022-01082-y (PMC9666995; doi:10.1186/s12912-022-01082-y)
Supplement: Supplementary file 1 — Additional file 1: Supplement Table1. Saturation grid. [file 12912_2022_1082_MOESM1_ESM.docx]

Supplement table1: saturation grid

| categories and subcategories | interviews | | | | | | | | | | | | | | | |
| --- | --- | --- | --- | --- | --- | --- | --- | --- | --- | --- | --- | --- | --- | --- | --- | --- |
|  | P1 | P2 | P3 | P4 | P5 | P6 | P7 | P8 | P9 | P10 | P11 | P12 | P13 | P14 | P15 | P16 |
| Categories one: The onset of a new chapter: from avoidance to relapse | * | * | * | * | * | * | * | * | * | * | * | * | * | * | * | * |
| Facing emerging developments | * | * | * | * | * | * | * | * | * | * | * | * | * | * |  | * |
| Empty the care area | * | * | * | * | * | * | * | * | * | * | * | * | * | * |  |  |
| Re-orientation and gradual care | * | * | * | * | * | * | * | * | * | * | * | * | * | * | * |  |
|  |  |  |  |  |  |  |  |  |  |  |  |  |  |  |  |  |
| Categories Two: Burnout | * | * | * | * | * | * | * | * | * | * | * | * | * | * | * | * |
| Mental pressure due to vulnerability | * | * | * | * | * | * | * | * | * | * | * | * | * | * |  | * |
| Physical injury | * | * | * | * | * | * | * | * | * | * | * | * | * | * |  |  |
| Stress caused by injury | * | * | * | * | * | * | * | * | * | * | * | * | * | * | * |  |
|  |  |  |  |  |  |  |  |  |  |  |  |  |  |  |  |  |
| Categories three: The vortex of moral distress | * | * | * | * | * | * | * | * | * | * | * | * | * | * | * | * |
| Spiritual suffering | * | * | * | * | * | * | * | * | * | * | * | * | * | * | * | * |
| Descendant helplessness | * | * | * | * | * | * | * | * | * | * | * | * | * | * | * | * |
|  |  |  |  |  |  |  |  |  |  |  |  |  |  |  |  |  |
| Categories four: social stigma | * | * | * | * | * | * | * | * | * | * | * | * | * | * | * | * |
| Perceived stigma | * | * | * | * | * | * | * | * | * | * | * | * | * | * | * |  |
| Perceived discrimination (feeling ignored) | * | * | * | * | * | * | * | * | * | * | * | * | * | * |  | * |
| Perceived rejection | * | * | * | * | * | * | * | * | * | * | * | * | * | * |  |  |
|  |  |  |  |  |  |  |  |  |  |  |  |  |  |  |  |  |
| Categories five: The difficulty in breaking the transmission chain | * | * | * | * | * | * | * | * | * | * | * | * | * | * | * | * |
| Physical separation difficulties | * | * | * | * | * | * | * | * | * | * | * | * | * | * | * | * |
| Difficulty implementing an individual protection strategy | * | * | * | * | * | * | * | * | * | * | * | * | * | * | * |  |
| Gradual decline in observing protocols over time | * | * | * | * | * | * | * | * | * | * | * | * | * | * |  | * |
|  |  |  |  |  |  |  |  |  |  |  |  |  |  |  |  |  |
| Categories six: care inhibitors related to patient and family | * | * | * | * | * | * | * | * | * | * | * | * | * | * | * | * |
| family-related care Barriers | * | * | * | * | * | * | * | * | * | * | * | * | * | * | * | * |
| patient-related care Barriers | * | * | * | * | * | * | * | * | * | * | * | * | * | * | * | * |
|  |  |  |  |  |  |  |  |  |  |  |  |  |  |  |  |  |
| Categories seven: Lack of support: crisis home care agencies in crisis | * | * | * | * | * | * | * | * | * | * | * | * | * | * | * | * |
| Crisis mismanagement | * | * | * | * | * | * | * | * | * | * | * | * | * | * |  |  |
| Lack of supervisor competence | * | * | * | * | * | * | * | * | * | * | * | * | * | * | * | * |
| Lack of information support: Lack of comprehensive training | * | * | * | * | * | * | * | * | * | * | * | * | * | * | * | * |
| Lack of financial and legal support: Compensation for inefficient services | * | * | * | * | * | * | * | * | * | * | * | * | * | * | * | * |
| Lack of logistical support: drug and equipment shortages | * | * | * | * | * | * | * | * | * | * | * | * | * | * | * | * |
| Lack of support for efficient manpower: Human resource mismanagement | * | * | * | * | * | * | * | * | * | * | * | * | * | * | * | * |
